# *Lactobacillus plantarum *(VR1) isolated from an Ayurvedic medicine (Kutajarista) ameliorates *in vitro *cellular damage caused by *Aeromonas veronii*

**DOI:** 10.1186/1471-2180-11-152

**Published:** 2011-06-27

**Authors:** Himanshu Kumar, Ashraf Y Rangrez, Kannayakanahalli M Dayananda, Ashwini N Atre, Milind S Patole, Yogesh S Shouche

**Affiliations:** 1 Molecular Biology Unit, National Centre for Cell Science, Ganeshkhind, Pune, Maharashtra, India; 2 Department of Pharmacy and Medicine, University of Picardie Jules Verne, Rue des Louvels, F-80037, Amiens, France; 3 Department of Chemical and Biological Engineering, State University of New York at Buffalo, Buffalo NY 14260, USA; 4 Microbial Culture Collection, Hindustan Antibiotics Ltd. Complex, Pimpri, Pune 411 018, Maharashtra, India

## Abstract

**Background:**

*Lactobacillus plantarum *is considered as a safe and effective probiotic microorganism. Among various sources of isolation, traditionally fermented foods are considered to be rich in *Lactobacillus *spp., which can be exploited for their probiotic attribute. Antibacterial property of *L. plantarum *has been demonstrated against various enteric pathogens in both *in vitro *and *in vivo *systems. This study was aimed at characterizing *L. plantarum *isolated from Kutajarista, an ayurvedic fermented biomedicine, and assessing its antagonistic property against a common enteropathogen *Aeromonas veronii*.

**Results:**

We report the isolation of *L. plantarum *(VR1) from Kutajarista, and efficacy of its cell free supernatant (CFS) in amelioration of cytotoxicity caused by *Aeromonas veronii*. On the part of probiotic attributes, VR1 was tolerant to pH 2, 0.3% bile salts and simulated gastric juice. Additionally, VR1 also exhibited adhesive property to human intestinal HT-29 cell line. Furthermore, CFS of VR1 was antibacterial to enteric pathogens like *Pseudomonas aeruginosa, Staphylococcus aureus, Escherichia coli*, *Aeromonas veronii *and clinical isolates of *P. aeruginosa *and *E. coli*. Detailed study regarding the effect of VR1 CFS on *A. veronii *cytotoxicity showed a significant decrease in vacuole formation and detrimental cellular changes in Vero cells. On the other hand, *A. veronii *CFS caused disruption of tight junction proteins ZO-1 and actin in MDCK cell line, which was prevented by pre-incubation with CFS of VR1.

**Conclusions:**

This is the first study to report isolation of *L. plantarum *(VR1) from Kutajarista and characterisation for its probiotic attributes. Our study demonstrates the antagonistic property of VR1 to *A. veronii *and effect of VR1 CFS in reduction of cellular damage caused by *A. veronii *in both Vero and MDCK cell lines.

## Background

Lactic acid bacteria is now widely used as probiont for its multifactorial benefits to humans as well as to organisms like fish, poultry and other live stock. In addition to various sources of isolation [1-3], several recent studies have described the isolation and characterisation of probiotic microorganisms from traditionally fermented sources like Dongchimi, Kimchi, Meju, and Doenjang [4], and Kallappam batter, Koozh and Mor Kuzhambu [5]. Likewise, traditional Ayurvedic medicines might serve as a source and a reservoir of potential probiotic microbes. Nevertheless, there are very little efforts made in exploration of probionts from ayurvedic fermented sources. To the best of our knowledge, Kanjika is the only Ayurvedic source explored in detail for the isolation and *in vitro *characterisation of *Lactobacillus *spp. for probiotic attributes [6]. In this study, Kutajarista is used as a source for the isolation of potential probiotic isolates. Kutajarista is a well known polyherbal Ayurvedic formulation prepared traditionally by fermentation of the decoction of *Holarrhena antidysentrica *as the main constituent [7]. It is being prescribed for a number of chronic diseases like amoebic dysentery, piles, intestinal parasites infestation and other disorders like fever, indigestion, and malabsorption syndrome [8].

There are growing number of studies that show the ability of *Lactobacillus *spp. to antagonize various pathogens, like enterohemorrhagic *E. coli *[9,10], *Helicobacter pylori *[11], *Salmonella typhimurium *[12], *Shigella dysenteriae *[13], using *in vitro *and *in vivo *systems. Probiotic microorganisms like *Lactobacillus *spp. exert beneficial effects on epithelial cells by secreting bioactive and extracellular proteins. Moreover, the active fraction has been isolated and tested for its activity as immunomodulators and inhibitors for pathogenic microorganisms [14,15]. Some recent reports also suggest the restoration of barrier function in epithelial cells by probiotic treatment due to the strengthening of tight junctions [10,16]. Gene expression profiling of tight junction proteins demonstrated the effect of *L. plantarum *MB452 in strengthening of tight junction associated proteins in Caco2 cell line [17]. Additionally, immunolocalization studies on tight junction proteins like ZO-1, claudin and F-actin demonstrate preventive role of *L. sobrius *in enterotoxigenic effect of *E. coli *K88 [18].

Among the species of *Aeromonas, A. hydrophila*, *A. salmonicida *and *A. veronii *are considered as emerging human pathogens and have a potent role in various gastrointestinal disorders. Several clinical studies highlight the outbreak of *Aeromonas *spp. infection in diarrhoea [19-21]. *Aeromonas *spp. harbours at various ecological niche, making the transmission of this pathogen more susceptible to humans [22]. *A. veronii *(MTCC 3249), bacterial strain that is used in this study was first reported from a mosquito midgut and subsequently reported from drinking water supplies and other sources [23-25], possess multiple virulence attributes like haemolytic activity, plasmids, quorum sensing and type four secretion system. These virulent properties can be implicated in its role for toxin production and transfer of antibiotic resistance genes across and within the genera [26-29]. In addition to previously established virulence traits, *A. veronii *was found to be coding for aerolysin and type three secretion systems.

In the current study, we isolated and characterised potential probiotic microorganisms from an Ayurvedic formulation, Kutajarista. We identified one of our twelve isolates, VR1, homologous to *L. plantarum *as a promising candidate exhibiting tolerance to low pH, bile salts and simulated gastric juice conditions. Accompanying studies demonstrated that VR1 is adherent to human derived HT-29 cells and antagonises the growth of commonly known pathogens like *S. aureus, P. aeruginosa *and particularly *A. veronii*. We further demonstrated that vacuole formation, epithelial damage and cytotoxicity caused by *A. veronii *was reduced or ameliorated by VR1.

## Results

### VR1 isolated from Kutajarista exhibited strong probiotic attributes

Twelve isolates obtained after enrichment of Kutajarista in MRS broth were identified on the basis of 16S rRNA gene sequencing. One of the isolates showed maximum homology with *L. plantarum *based on 16S rRNA gene sequence [GenBank: HQ328838]. Its phylogenetic affiliation was deduced by comparing the homologous 16S rRNA gene sequences from NCBI and the phylogenetic tree is shown in additional file 1, Fig S1.

**Figure 1:**
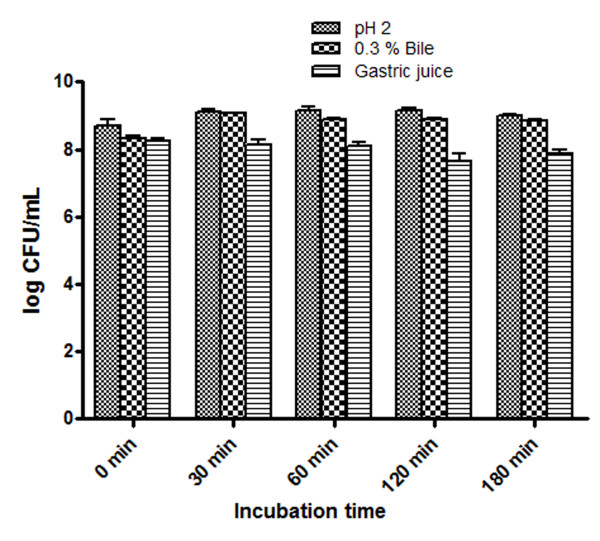
**Probiotic properties of VR1**. The chart representing the tolerance of VR1 to various physiological conditions of a) pH 2 b) 0.3% bile salts and c) simulated gastric juice, determined at various time points. Data is presented as mean of three independent experiments.

Acid, bile and gastric juice tolerance is considered to be the preliminary characteristics of any strain to claim its probiotic potential [2,30]. VR1 showed tolerance to low pH (pH 2.0), bile salt concentration of 0.3% and simulated gastric juice. There was a little increase of 0.3 Log (CFU/ml) during the course of incubation for 3 h, which further suggested that it can tolerate and remain viable at acidic pH 2.0 (Figure 1). In 0.3% bile, there was increase of 0.5 Log (CFU/ml) after 3 h of incubation and in simulated gastric juice tolerance test, a decrease of 0.4 Log (CFU/ml) on growth was observed. *L. plantarum *is known to be adherent to intestinal cell lines like Caco2 and HT-29. This study showed that VR1 was adherent to HT-29 cell line with the adhesion ratio of 6.8 ± 0.2%, which was in concordance with the earlier studies [31].

### CFS of VR1 antagonised the growth of enteric pathogens

Antagonistic activity of VR1 culture supernatant was examined using well-diffusion test against *S. aureus *(ATCC 6538P), *S. lutea *(ATCC 9341), *A. veronii *(MTCC 3249), *E. coli *(ATCC 8739), *P. aeruginosa *(ATCC 27853), *S. epidermidis *(ATCC 12228), and clinical isolates of *P. aeruginosa *(DMH 1), and *E. coli *(DMH 9). VR1 showed antimicrobial activity against all the tested microorganisms, with strong antibacterial activity against *A. veronii *with 22 mm inhibitory zone (Table 1).

**Table 1:** Antibacterial activity of VR1 against various pathogens

Test Organism	Zone of Inhibition (mm)^1, 2^
*Staphylococcus aureus *(ATCC6538P)	18
*Sarcina lutea *(ATCC 9341)	17
*Escherichia coli *(ATCC 8739)	20
*Pseudomonas aeruginosa *(ATCC27853)	18
*Staphylococcus epidermidis *(ATCC12228)	16
*Pseudomonas aeruginosa *(DMH 1)	16
*Escherichia coli *(DMH 9)	16
*Aeromonas veronii*(MTCC 3249)	22

^1^Diameter of the well 7 mm. ^2^Values shown represent the mean of three replicates

### Vacuole formation by *A. veronii *on Vero cells were moderated by VR1 CFS

**Figure 2:**
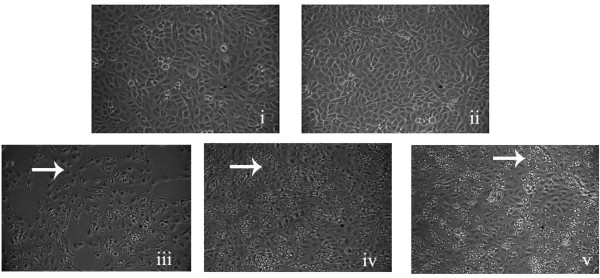
**Effect of VR1 culture supernatant on reducing the vacuolation caused by* A. veronii***. A confluent monolayer of Vero cells treated with culture supernatant, i) control, ii) VR1, iii) *A. veronii*, iv) VR1 and *A. veronii *v) *A. veronii *on Vero cells pre-incubated with VR1 supernatant for 6 h. It is evident that the vacuole formation was decreased when Vero cells were pre-incubated with VR1 supernatant. Arrow indicates vacuolation in Vero cells after treatment with *A. veronii *culture supernatant.

Sensitivity of Vero cells for *Aeromonas *cytotoxicity has been well documented [32-34]. Vero cells were treated with CFS of *A. veronii *and VR1, in 1:10 ratio in DMEM. Figure 2 revealed the formation of perinuclear vacuoles in more than 50% of cells and cell detachment was observed after five hours of incubation with *A. veronii *CFS; however, pre-incubation with VR1 supernatant for 6 h reduced the vacuole formation and cell detachment.

### Time lapse microscopy revealed delayed cytotoxic effects of *A. veronii *on Vero cells pre-incubated with VR1

**Figure 3:**
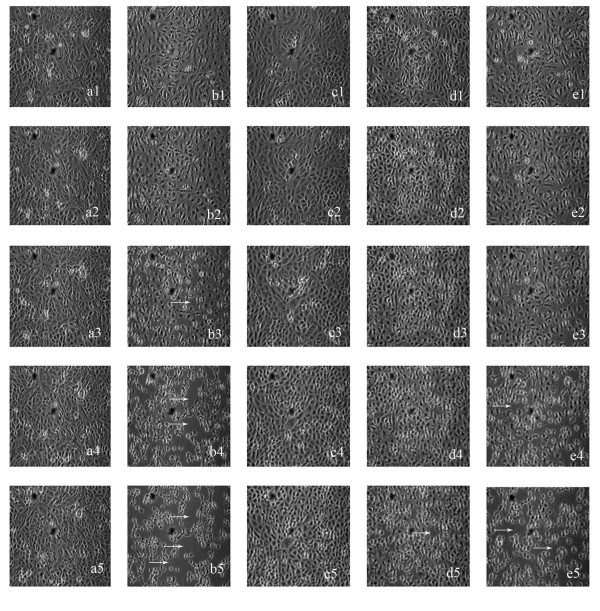
**Effect of VR1 *CFS *in delaying the cytotoxicity caused by *A. veronii***. Time lapse microscopic studies were carried out until 10 h incubation of Vero cells with different treatments of culture supernatant of *A. veronii *and VR1 in 1:10 ratio. We show here the representative images from the treatment of a) control b) *A. veronii *c) VR1 d) pre-incubation of VR1 for 6 h and then addition of *A. veronii *e) co-incubation of VR1 and *A. veronii*. Images a1-a5 represents the incubation time of 2, 4, 6, 8 and 10 h, respectively. Same denomination is followed for other treatments as well. Detachment of Vero cells can be observed from 6 h onwards in *A. veronii *treated cells. Arrow indicates cell detachment.

Time lapse microscopic images were taken at various time intervals for 10 h (Figure 3). Treatment with *A. veronii *supernatant in 1:10 ratio to media started showing acute cytopathic effect with cell detachment from the surface, after 6 h of incubation. Alteration in Vero cells was followed by a change from normal spindle shaped to round swollen morphology with an extensively altered cytoplasm and gradual destruction of the monolayer. However, these cytopathic effects were delayed by 2 h, where *A. veronii *supernatant was co-incubated with VR1 supernatant. Vero cells pre-treated for 6 h with VR1 supernatant showed marked reduction in the cytotoxicity caused by *A. veronii*, and only few cells were detached even after 10 h of incubation.

### VR1 prevented disruption of ZO-1 and F-actin caused by *A. veronii*

**Figure 4:**
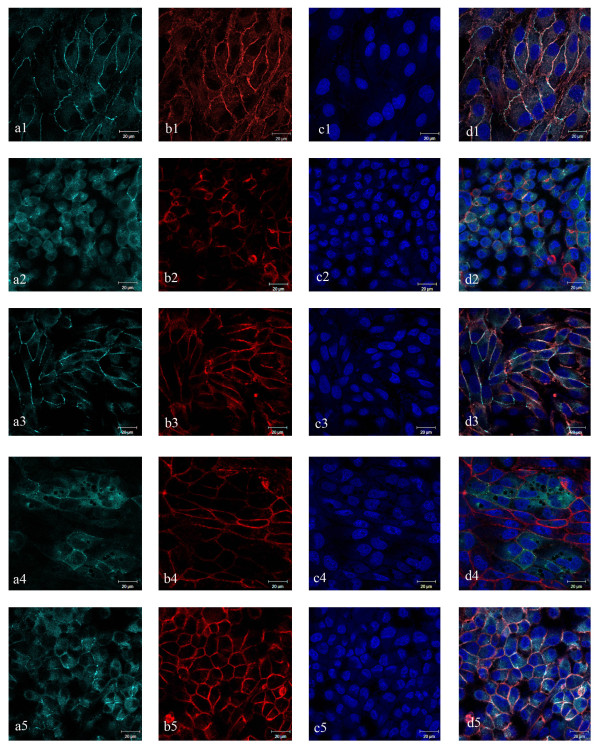
**Prevention of membrane damage caused due to *A. veronii* by pre-incubated with CFS of VR1**. Epithelial damage observed by immunofluorescence of tight junction proteins ZO-1 and F-actin in MDCK cell line. a) ZO-1 b) Actin c) DAPI d) Merged images for different treatment groups: 1) control, 2) *A. veronii *3) VR1 4) co-incubation of VR1 with *A. veronii *5) pre-incubation of VR1 with *A. veronii*. Pre-incubation of VR1 prevents epithelial damage due to *A. veronii *as observed in the merged image. Scale denotes 20 μm in all images.

Immunofluorescence for tight junction protein ZO-1, revealed continuous and circumferential ZO-1 distribution in MDCK cells treated with VR1 CFS (Figure 4a3) similar to control cells (Figure 4a1). However, fragmented, diffused and punctated pattern of ZO-1 distribution was observed in case of cells treated with *A. veronii *supernatant (Figure 4a2). Pre-incubation of MDCK cells with VR1 for 6 h prior to *A. veronii *infection prevented these changes and showed clear delineation of cellular borders in a belt like manner (Figure 4a5). Treatment with *A. veronii *supernatant led to disorganisation of actin filaments and nuclear condensation was also observed (Figure 4b2 &4c2). However, pre-incubation of cells with VR1 supernatant maintained the cellular morphology comparable to control cells. In both the treatments i.e. VR1 CFS, and *A. veronii *CFS treatment on cells that were pre-incubated with CFS of VR1, actin filaments were present in high density at the apical perijunctional regions, encircling the cells in a belt like manner (Figure 4b3 &4b5). However, co-incubation of *A. veronii *and VR1 supernatant (Figure 4a4 to 4d4) led to the loss of membrane architecture with loss of fluorescence of ZO-1 and actin, as observed in *A. veronii *treatment group.

**Figure 5:**
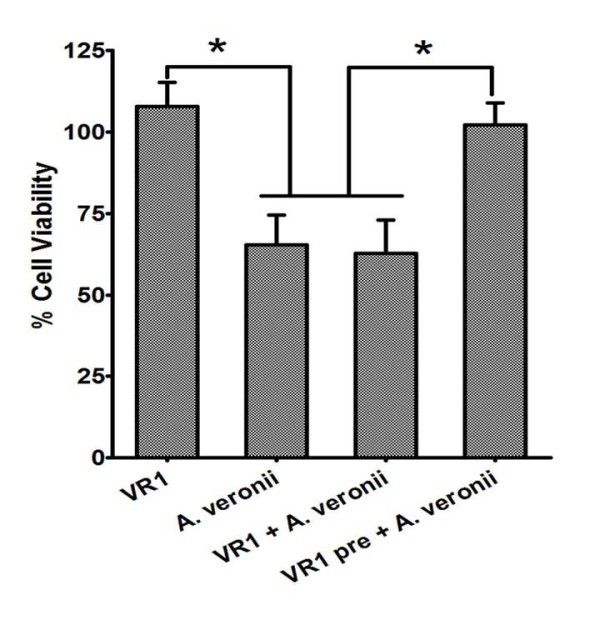
**Effect of VR1 culture supernatant in preventing the loss of cell viability caused due to *A. veronii***. MTT assay was performed to quantify percentage cell viability with treatment of supernatant of *A. veronii *and VR1, in 1:10 ratio. Cell viability graph demonstrates that the pre-incubation with VR1 supernatant for 6 h significantly increased the cell viability. Statistical significance was determined by two tailed student's *t-*test (n = 3 ± SEM, *p < 0.05).

### CFS of VR1 significantly lowered cytotoxicity induced by *A. veronii*

The cytotoxic effect of *A. veronii *CFS was confirmed by MTT assay, which essentially checks cell viability (Figure 5). Cell viability was reduced to 60% in Vero cells treated with *A. veronii *supernatant for 10 h. Interestingly, Vero cells when pre-incubated with VR1 CFS for 6 h followed by 10 h of treatment with *A. veronii *CFS showed no loss of cell viability. Similarly, VR1 CFS treatment did not show any detrimental effects on cells with no loss in cell viability. However, co-incubation of VR1 and *A. veronii *supernatant was not effective in preventing cytotoxicity caused by *A. veronii*.

## Discussion

Kutajarista is an Ayurvedic formulation prescribed for the treatment of dysentery, piles etc. Initial characterisation of bacterial diversity of Kutajarista by the 16S rRNA gene clone library [GenBank: HQ875575-HQ875614] provided evidence about the richness of *Lactobacillus *spp. in the preparation of ayurvedic medicine. Therefore, the current study was aimed at characterization of probiotic and antibacterial properties of *L. plantarum *VR1 isolated from Kutajarista, against a known cytotoxic and virulent strain, *A. veronii*. Previously, it has been reported that *L. delbrueckii, L. lactis *and *L. mesenteroides *can prevent cellular damage caused by *A. salmonicida*, a fish pathogen [35,36]. Here we report that VR1 possess strong probiotic properties and abrogated the cytotoxicity of *A. veronii *MTCC 3249, an isolate from mosquito midgut. To the best of our knowledge this is the first report of the preventive role of CFS from VR1 in cellular and epithelial damage caused by *A. veronii*.

Traditionally fermented products are rich source of *Lactobacilli*, which can be exploited for their probiotic potential. Indian fermented foods like Kallappam, koozh and Mor Kuzhambu were reported as a source of potential probiotic *Lactobacillus *spp. and which is useful as biopreservative [5]. Ayurveda is traditionally practised medicinal science for many centuries and medicines are prepared from herbs. However, very little efforts have been made in utilizing these preparations as a source of probionts. There is only major study which reported the isolation and charactarisation of seventeen *Lactobacillus *spp. from Kanjika, an Ayurvedic formulation, for probiotic attributes [6]. In the present study, we used Kutajarista, an Ayurvedic herbal decoction, for isolation of potential probiont. VR1 showed highest homology to *L. plantarum *and exhibited probiotic characteristics such as tolerance to acidic pH, bile salts and simulated gastric juice. VR1 also showed adherence to intestinal cell line HT-29, which is one of the essential prerequisites for a probiotic microorganism. All these features indicate this strain of *L. plantarum *as a potential probiont. A recent report by Anderson et al. [37] suggests that *L. plantarum *has better probiotic characteristics and it also reduces enteropathogenic effect of *E. coli *as compared to commercial strains like *L. rhamnosus*. Moreover, *L. plantarum *has been reported to inhibit pathogens in *in vitro *and *in vivo *systems [9,13]. On the same lines, *L. plantarum *isolated from Kutajarista showed inhibition of the tested type strains and clinical isolates of *P. aeruginosa *and *E. coli*. Interestingly VR1 also prevented the growth of *A. veronii*, for which virulent attributes have already been established [26-28].

The pathogenicity of genus *Aeromonas *is multifactorial and is attributed to factors such as; cytotoxin, aerolysin, hemolysin, adhesins and secretory systems. Apart from other virulence factors which may contribute to the pathogenesis of *A. veronii*, here we report the presence of type three secretion system and aerolysin (additional file 2, Fig S2), putatively involved in secretion of virulence factors to the host cell and haemolytic activity respectively. Our previous studies have also demonstrated that *A. veronii *MTCC 3249 is multi-drug resistant, and harbours three uncharacterised plasmids and one of the plasmids codes for functional type four secretion system [26,28,29].

After establishing the fact that *A. veronii *was cytotoxic to mammalian cells and harbours many virulence factors, our next goal was to observe the protective or preventive role of VR1 in *A. veronii *infection. We used CFS of VR1 to examine its efficacy in amelioration of cytotoxicity caused by *A. veronii *supernatant. We observed high level of vacuole formation as an indication of cytotoxicity and morphological changes in Vero cells. Earlier, in an enterohaemorrhagic *E. coli *infection model, it was shown that pre-incubation with *L. plantarum *abolished the cytotoxicity caused by enteropathogenic strain [10]. To test whether VR1 had similar effects, we studied the time dependent effects of CFS of *A. veronii*, VR1, in combination or treatment of *A. veronii *on VR1 pre-incubated cells. We found that pre-incubation of Vero cells with VR1 CFS delayed cytotoxicity, which was induced by *A. veronii*. Vacuolating cytotoxic factor from *A. veronii *was earlier reported to cause cell death [38].

Tight junction disruption is considered to be one of the indicators of morphological damage caused due to cytotoxicity. MDCK cell line infected with *V. cholerae *cytotoxin and *S. typhimurium *showed a clear indication of epithelial barrier dysfunction by disruption of tight junction [39,40]. In fish, pre-incubation with prospective probiont *L. delbrueckii *sub sp. *lactis *could prevent epithelial damage caused by *A. salmonicida *[36]. To investigate the effect of CFS derived from VR1, and *A. veronii *on epithelial barrier, we selected MDCK cell line over Caco2 cell line because it exhibits similar epithelial characteristics like formation of uniform columnar epithelia, tight junction, and it has an advantage of a short culture period of 5-7 days in comparison to Caco2 which has 21 days of growth period [41-43]. We found that *A. veronii *indeed caused epithelial damage by disruption of ZO-1 and F-Actin in MDCK cell line, which was prevented by pre-incubation with VR1 supernatant for 6 h, whereas co-incubation was not able to restore the epithelial integrity. ZO-1 is a cytoplasmic protein which interacts directly with F-Actin and is very important in structural and functional organisation of tight junction. In this study, microscopic observation of cellular damage is well supported by immunolocalization of ZO-1 and F-Actin, which give clear evidence of VR1 in ameliorating the epithelial damage caused by *A. veronii*. This finding is consistent with earlier report that, *L. rhamnosus *GG treatment ameliorated the redistribution of ZO-1 and claudin in MDCK cell line caused by enterohemorrhagic *E. coli *[16]. In another study, incubation with CFS of *B. lactis *420 has been shown to increase the intestinal epithelial integrity against enteropathogenic *E. coli *(EPEC) [44].

Cell viability assessed by MTT assay revealed that VR1 CFS treatment was not detrimental to cells and there was no loss in viability when pre-incubated with VR1 CFS. On the other hand, co-incubation could not prevent the loss in cell viability caused by *A. veronii*. Pre-incubation of Caco2 with p40 and p75 isolated from the soluble protein of *L. rhamnosus *GG, abrogated the disruptive effect of H_2_O_2 _on tight junctions of Caco2 cells [45]. The protective effect of soluble proteins was shown to be by activation of MAP kinase and PKC dependent signalling pathways. One more study (Parassol et al., [46]) documented that pre-incubation of *L. casei *with T84 cells could abolish the invasion and adhesion of EPEC. On these lines, we speculate, pre-incubation of mammalian cells with CFS of *Lactobacilli *sp. initiates cellular signalling which either inhibits or upregulate tight junction proteins that may get damaged by entero pathogens.

In view of the increasing prevalence of *Aeromonas *spp. in food products, this study assumes significance of its application of *L. plantarum *as a potential probiotic microorganism. The findings also suggest that the regular usage of probiotic microorganisms in food preparations can prevent the cytotoxicity or manifestation of pathogenicity in future encounter with pathogens. Further in depth studies will be necessary to understand the preventive role of VR1 in *invivo *model for *A. veronii *infection and to identify its active component which may be used as potential preventive cure against gastro-intestinal infection.

## Conclusions

To the best of our knowledge, this is the first report of isolation of potential probiotic isolate, *L. plantarum *VR1 from Kutajarista, an ayurvedic fermented medicine. CFS of VR1 possesses strong antibacterial property against *A. veronii *and reduces its cytotoxic effects in MDCK and Vero cell lines. Hence, *L. plantarum *can be an effective probiotic to prevent *Aeromonas *infection as well, as it has been proposed for some other enteric pathogens.

## Methods

### Bacterial strains and growth conditions for mammalian cells

The bacterial strains used in this study are *A. veronii *MTCC 3249, *L. plantarum *(VR1) NCIM 5395 and *E. coli *DH5α. Strains used for antimicrobial study were *S. aureus *(ATCC 6538P), *Sarcina lutea *(ATCC 9341), *E. coli *(ATCC 8739), *P. aeruginosa *(ATCC 27853), *S. epidermidis *(ATCC 12228), clinical isolates of *P. aeruginosa *(DMH 1), *E. coli *(DMH 9). All the above mentioned type strains, *A. veronii *and *E. coli *were maintained in Luria Bertani (LB) medium at 37°C. VR1 was grown in Man Rogosa Sharpe (MRS) medium (Himedia Laboratories, Mumbai, India) at 37°C. Overnight grown cultures of *A. veronii *and VR1 were inoculated into 5 ml of LB and MRS medium respectively, at 37°C with shaking at 200 rev min^-1^. Cell-free supernatant was prepared by centrifugation (10,000 g for 2 min at 4°C) followed by filtration of the supernatants through a 0.22 μm pore size membrane filter (Millipore, India). The filtrates were either refrigerated before use or used immediately. HT-29 (Human colon adenocarcinoma), Vero (African green monkey Kidney) and MDCK (Madin-Darby Canine Kidney) epithelial cell lines, were purchased from the animal cell repository of National Centre for Cell Science, India and maintained in DMEM supplemented with 10% Fetal Calf serum and 1 × Penicillin-streptomycin (Invitrogen, Carlsbad CA).

### Isolation and identification of *Lactobacillus spp*. from Kutajarista

Several samples of Kutajarista, (an Ayurvedic fermented decoction) were taken at initial days of fermentation. A number of lactic acid bacterial strains were isolated (serial dilution with saline) in MRS plate and incubated at 37°C for 2-3 days. All isolated strains were subsequently propagated in MRS broth and were stored in 40% glycerol in -80°C. Molecular identification was carried out by amplification and sequencing of ~1.5 Kb partial sequence of 16S rRNA gene by using Eubacteria specific 16F27 (5'-CCA GAG TTT GAT CMT GGC TCA G-3') and 16R1488 (5'- CGG TTA CCT TGT TAC GAC TTC ACC -3') [23]. The 16S rRNA gene sequence for the strain VR1 was submitted to Genbank with accession number HQ328838. VR1 showed 99% homology with *Lactobacillus plantarum *and its phylogenetic affiliation was deduced by neighbour joining method in MEGA 4.0.

### *In vitro *characterisation of VR1 for probiotic attributes

MRS broth was used to simulate the acidic condition of intestine by adjusting the pH of the broth to pH 2. For bile tolerance test, MRS broth was supplemented with 0.3% bile salts (Oxgall, Himedia, India). Simulated intestinal fluid was prepared to assess passage through the upper gastrointestinal tract. The composition of simulated gastric juice was 1.28 g NaCl, 0.239 g KCl, 6.4 g NaHCO_3_, 0.3% bile salts, 0.1% (w/v) pancreatin (Hi media Labs) per litre of distilled water and the pH to 7.5 adjusted by adding HCl [30]. For all the tolerance tests, 5 ml overnight grown *Lactobacillus *strains were collected by centrifugation and washed twice with 4 ml of PBS and inoculated (at 10^9 ^CFU/ml) in MRS broth with modifications for acid, bile and gastric juice tolerance medium mentioned above. Then the number of viable VR1 cells was determined by serial dilution and plate-count method.

### Antimicrobial activity of VR1

The antimicrobial activity of VR1 was determined by well diffusion assay as described by Chiu et al. [47]. Bacterial strains included in this study were *S. aureus *(ATCC 6538P), *S. lutea *(ATCC 9341), *A. veronii *(MTCC 3249), *E. coli *(ATCC 8739), *P. aeruginosa *(ATCC 27853), *S. epidermidis *(ATCC 12228), and clinical isolates *P. aeruginosa *(DMH 1), and *E. coli *(DMH 9). These bacterial isolates were grown overnight in LB broth and further diluted to 10^7 ^CFU/mL and spread on LB agar plates. One hundred microliters of filtered spent CFS of VR1 were pipetted into the well on nutrient agar and then plates were incubated at 37°C for 12-14 h. The diameters of the zone of inhibition were measured.

### Adhesion assay of VR1

Adhesion of VR1 was performed using HT-29 cells as described earlier with little modification [2,4]. Briefly, monolayers of HT-29 were used at the late confluence with change of media every 2 days. HT-29 monolayers were washed twice with sterile PBS. The Antibiotic free media was added to each well and incubated for 12 h. For each adhesion assay, 1 ml of VR1 suspension (the final concentration of bacteria was 10^9 ^CFU/ml) was mixed with 1 ml of DMEM and added to different wells. The plates were incubated at 37°C for 1.5 h in the presence of 5% CO_2_. After incubation, monolayer was washed with sterile PBS. One ml of 0.2% trypsin was added to each well and incubated for 15 min at Room temperature (RT). The cell suspension was plated on MRS agar by serial dilution using saline. Results were interpreted as percentage adhesion, the ratio between adherent bacteria and added bacteria per well. Three independent experiments were carried out in duplicate.

### DNA manipulations, Hybridization, PCR and Sequencing

*A. veronii *genomic DNA was extracted using a standard method [48]. Primer pairs and PCR conditions used for amplification of aerolysin, hemolysin and *ascV *genes are given in additional file 3, Table S1. Dot blot hybridization was performed with *^α^*^32^P labelled dATP using Amersham Megaprime DNA labelling system. Transfer of DNA to nylon membrane, hybridization conditions, and visualization were according to the manufacturer's protocol. DNA sequencing was carried out on 3730 DNA Analyzer with an ABI PRISM BigDye Terminator cycle sequencing kit (Applied Biosystems). The partial sequence of *A. veronii ascV *gene was submitted to Genbank with accession number HQ602648.

### Assessment of vacuole formation by light microscopy

Bacterial cultures were grown and CFS was prepared as described above and processed for vacuolation assay as described previously [33,49] with slight modifications. Briefly, Vero cells were seeded in six well tissue culture plate with cell density of 1 × 10^5 ^cells/ml. The cells were allowed to settle, attach and grow for 24 h prior to use. 100 μl of filter sterilized *A. veronii*, and VR1 CFS, were added to the respective wells, mixed gently and incubated for 5 h before taking the images. One of the wells was pre-incubated with VR1 supernatant for 6 h before the addition of *A. veronii *supernatant. Vacuolation was observed by Phase contrast microscopy (Nikon 2000, Japan). Images were taken under 20 × objective and were analysed using image pro software (Media Cybernetics, Inc, Bethesda, MD).

### Time lapse microscopic analysis of cytotoxic effect

For photomicroscopy, Vero cells were seeded in six well tissue culture plate with the density of 1 × 10^5 ^cells/well. After 24 h of incubation for cell attachment, cells were treated with bacterial supernatant with a concentration of 1:10 to the culture media; one of the wells was pre-incubated with probiotic supernatant for 6 h prior to the treatment with *A. veronii *supernatant. Other treatment groups were same as described above. Live imaging was performed and images were captured at the intervals of 30 min using NIKON TE 2000 under 20 × objective. Images were analysed by Image pro from media analytica.

### Analysis of ZO-1 and F-actin distribution by confocal microscopy

MDCK cells were seeded on the cover slips into 24 well tissue culture plates and incubated with 1:10 ratio of CFS of *A. veronii *and VR1. The tissue-culture plates were incubated in 5% CO_2 _atmosphere at 37°C for 10 h with *A. veronii *supernatant, or with VR1, in other group three wells were pre-incubated with VR1 for 6 h before addition of *A. veronii *supernatant. The immunofluorescence staining protocol was adopted from Johnson-Henry, [16]. Briefly, MDCK cell monolayers were rinsed with PBS, followed by fixation and permeabilization with 0.1% triton X-100 for 5 min at RT. Cells were incubated in 5% (vol/vol) bovine serum in PBS for 1 h at RT and then incubated with primary mouse anti-ZO-1 (339100, Invitrogen, molecular probes, USA) for 1 h. Unbound primary antibodies were rinsed and removed by washes with PBS, cells were incubated with secondary ALEXAfluor 633 goat anti-mouse IgG (1:50 dilution; Molecular Probes) and Rhodamine-phalloidin (1: 100 dilution, R-415, Molecular probes) for 1 h at RT. Host cell nuclei were counterstained with 300 nM 4^'^,6-diamidino-2-phenylindole dilactate (DAPI) (Molecular Probes) in PBS for 5 min. Monolayers were thoroughly rinsed with PBS, mounted on slides and examined under confocal laser scanning microscope at 1-μm intervals (Zeiss LSM510; Zeiss, Germany).

### Cytotoxicity assay

MTT reduction assay was performed to determine the effect of CFS of *A. veronii *on Vero cell viability. This method was adopted from Couto et al. [50] with little modifications. 10 μl of CFS of VR1 and *A. veronii *were added to a final concentration of 1: 10 in culture media of Vero cells cultivated in 96-well tissue culture plates. The tissue-culture plates were incubated in 5% CO_2 _atmosphere at 37°C for 10 h. Monolayers was examined after 10 h of incubation for cytotoxic effect. 20 μl of MTT solution (5 mg ml^-1^) was added to every well. After incubation for 3 h at 37°C, the media was removed and precipitated formazan was dissolved with 100 μl of DMSO. The absorbance was measured at 570 nm using Micro-plate reader (Multiskan Ascent V1.24). The cell viability was expressed as the mean of percentages of treated and untreated monolayers. Experiments were performed in triplicate.

## Competing interests

The authors declare that they have no competing interests.

## Authors' contributions

HK, AYR, YSS and MSP designed this study. HK and AYR were involved in standardization of the experimental conditions. HK was involved in acquisition of the data. HK, AYR, KMD and ANA analyzed and interpreted the data. HK wrote the first draft of the manuscript, other authors edited and revised the manuscript. All authors read and approved the final manuscript.

## Supplementary Material

Additional file 1 **Figure S1. Phylogenetic relationships of VR1 to reference strains of the genus *Lactobacillus***. The unrooted phylogenetic tree was drawn using 1320 nucleotides of 16S rRNA gene sequence using the neighbour-joining method in MEGA software. The bar represents distance values calculated in MEGA and values at nodes represent bootstrap percentages. Bootstrap values less than 50% is not shown. Click here for file

Additional file 2 **Figure S2. Detection of *Hemolysin *and *Aerolysin *genes in *A. veronii***. (A) Dot Blot of genomic DNA with Hemolysin gene as a probe. Lane 1- *A. hydrophila *ATCC 3484; Lane 2- *A. hydrophila *ATCC 7966; Lane 3- *A. veronii *(B) Lane 1, *A. veronii *aerolysin partial gene; M- molecular weight marker (Invitrogen). (C) Lane 1, *A. veronii *haemolysin partial gene; Lane 2, *A. hydrophila *ATCC 3484; Lane 3, *A. hydrophila *ATCC 7966, M- molecular weight marker (Invitrogen). Click here for file

Additional file 3 **Table S1. Primer combinations used for detecting the virulence gene determinants in *A. Veronii*. **Primer pairs used for amplification of aerolysin, hemolysin and *ascV *genes. Click here for file
