# Supplementary figures and images for: Lactobacillus plantarum (VR1) isolated from an Ayurvedic medicine (Kutajarista) ameliorates in vitro cellular damage caused by Aeromonas veronii
Source: BMC Microbiol. 2011 Jun 27;11:152. doi: 10.1186/1471-2180-11-152 (PMC3145568; doi:10.1186/1471-2180-11-152)

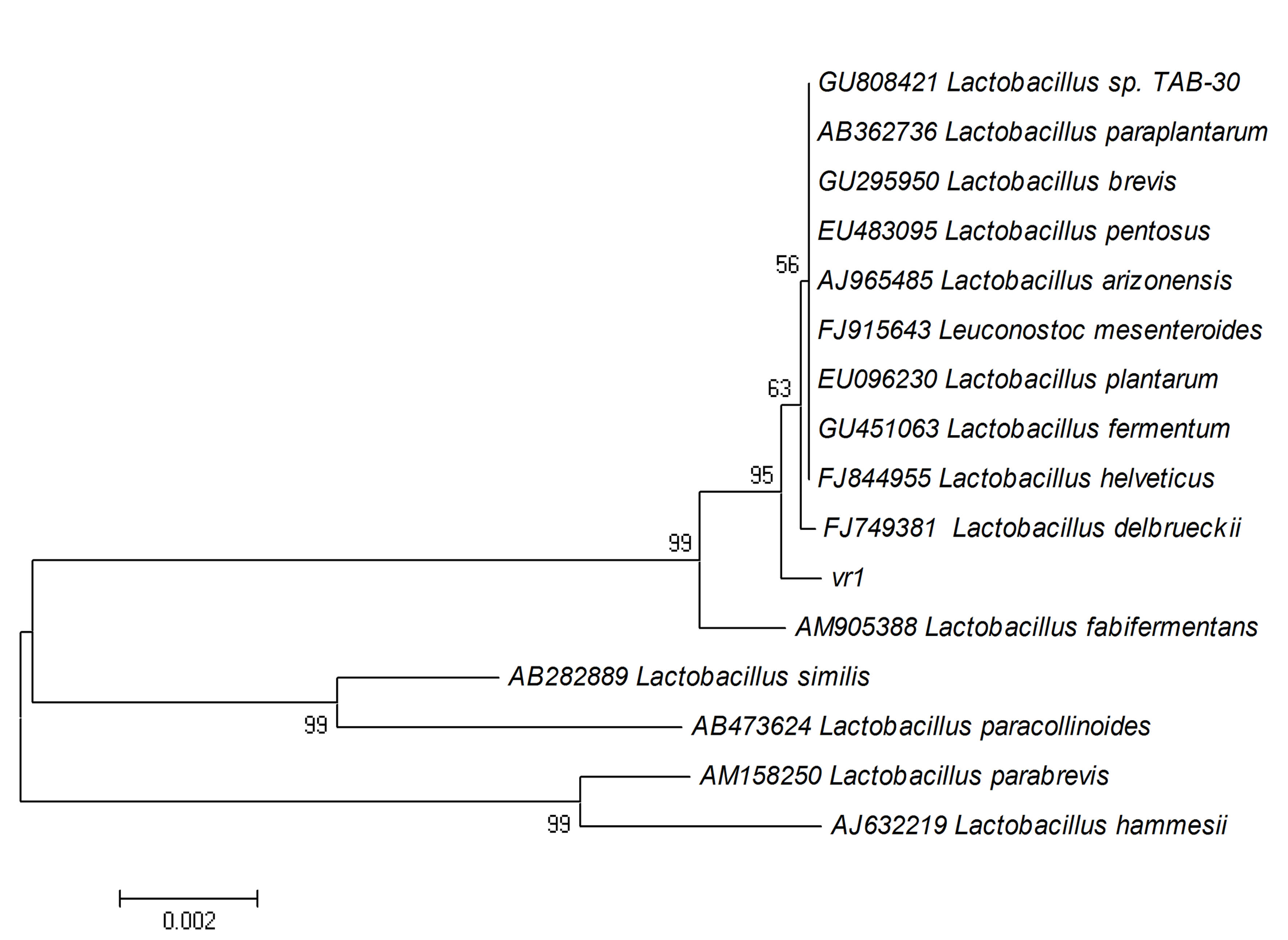

Supplement: Additional file 1 — Figure S1. Phylogenetic relationships of VR1 to reference strains of the genus Lactobacillus. The unrooted phylogenetic tree was drawn using 1320 nucleotides of 16S rRNA gene sequence using the neighbour-joining method in MEGA software. The bar represents distance values calculated in MEGA and values at nodes represent bootstrap percentages. Bootstrap values less than 50% is not shown. [file 1471-2180-11-152-S1.JPEG]

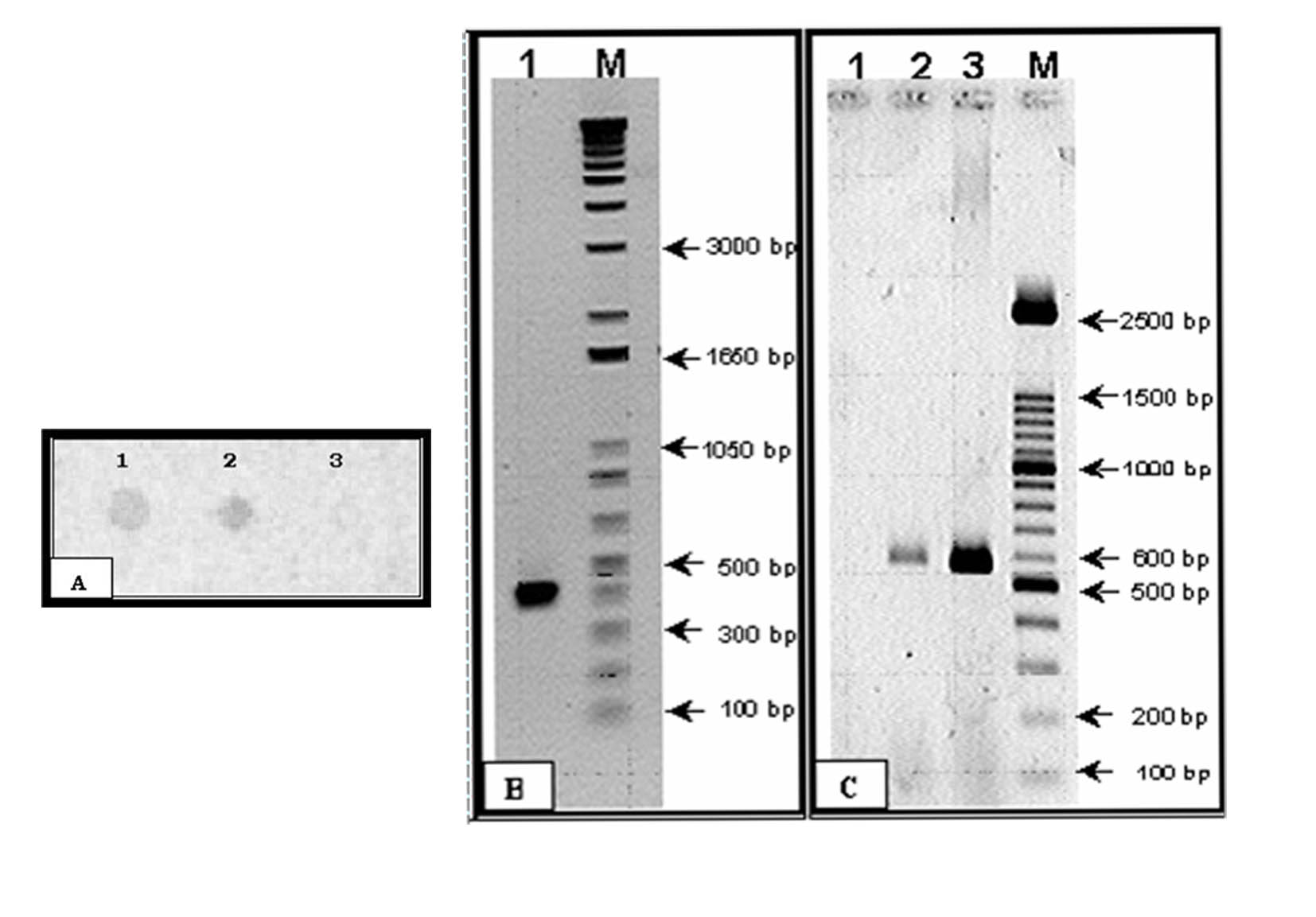

Supplement: Additional file 2 — Figure S2. Detection of Hemolysin and Aerolysin genes in A. veronii. (A) Dot Blot of genomic DNA with Hemolysin gene as a probe. Lane 1- A. hydrophila ATCC 3484; Lane 2- A. hydrophila ATCC 7966; Lane 3- A. veronii (B) Lane 1, A. veronii aerolysin partial gene; M- molecular weight marker (Invitrogen). (C) Lane 1, A. veronii haemolysin partial gene; Lane 2, A. hydrophila ATCC 3484; Lane 3, A. hydrophila ATCC 7966, M- molecular weight marker (Invitrogen). [file 1471-2180-11-152-S2.JPEG]
