# Supplementary material for: Lactobacillus plantarum (VR1) isolated from an Ayurvedic medicine (Kutajarista) ameliorates in vitro cellular damage caused by Aeromonas veronii
Source: BMC Microbiol. 2011 Jun 27;11:152. doi: 10.1186/1471-2180-11-152 (PMC3145568; doi:10.1186/1471-2180-11-152)
Supplement: Additional file 3 — Table S1. Primer combinations used for detecting the virulence gene determinants in A. Veronii. Primer pairs used for amplification of aerolysin, hemolysin and ascV genes. [file 1471-2180-11-152-S3.DOC]

**Supplementary Table 1:**

**Primer combinations used for detecting the virulence gene determinants in *A. veronii***

| **Sl. No.** | **Gene Targeted** | **Primer**  **Name** | **Sequence (5’-3’)** | **Product Length** |
| --- | --- | --- | --- | --- |
| 1 | *ascV* | ascV-F and ascV-R | TAARCAGATGAGTATCGATGG  GAGACSCGGGTGACGATAAT | 331 bp |
| 2 | *Aerolysin* | A1 and  A2 | GCCTGAGCGAGAAGGT  CAGTCCCACCCACTTC | 416 bp |
| 3 | *Hemolysin* | H1 and  H2 | GGCCGGTGGCCCGAAGATACGGG  GGCGGCGCCGGACGAGACGGG | 597 bp |
